# Optimisation of an Advanced Oxidation Protein Products Assay: Its Application to Studies of Oxidative Stress in Diabetes Mellitus

**DOI:** 10.1155/2015/496271

**Published:** 2015-05-14

**Authors:** Emma L. Taylor, Kenneth R. Armstrong, David Perrett, Andrew T. Hattersley, Paul G. Winyard

**Affiliations:** ^1^University of Exeter Medical School and NIHR Exeter Clinical Research Facility, University of Exeter, Exeter EX1 2LU, UK; ^2^Barts and the London School of Medicine and Dentistry, Queen Mary University of London, London EC1M 6BQ, UK

## Abstract

Advanced oxidation protein products (AOPP) are reportedly elevated in the plasma of patients with a number of diseases, including diabetes mellitus, that involve oxidative stress. However, the accurate measurement of AOPP in human plasma is hampered by the formation of a precipitate following the addition of potassium iodide and glacial acetic acid according to the published assay procedure. Here we describe a modification of the AOPP assay which eliminates interference by precipitation and provides a robust, reliable, and reproducible protocol for the measurement of iodide oxidising capacity in plasma samples (intra-assay CV 1.7–5.3%, interassay CV 5.3–10.5%). The improved method revealed a significant association of AOPP levels with age (*p* < 0.05) and hypertension (*p* = 0.01) in EDTA-anticoagulated plasma samples from 52 patients with diabetes and 38 nondiabetic control subjects, suggesting a possible link between plasma oxidising capacity and endothelial and/or vascular dysfunction. There was no significant difference between AOPP concentrations in diabetic (74.8 ± 7.2 *μ*M chloramine T equivalents) and nondiabetic (75.5 ± 7.0 *μ*M chloramine T equivalents) individuals.

## 1. Introduction

The availability of plasma oxidative damage assays which are sensitive and robust is a limiting factor in high-throughput studies of human disease and ageing [1]. The advanced oxidation protein products (AOPP) assay is a widely published method to determine plasma oxidative stress, based on a spectrophotometric assay in a 96-well microplate format [2]. AOPP have been analysed in numerous diseases and are widely regarded as an easily measurable marker of oxidative stress [3–6].

Oxidative stress is a well-established feature of diabetes mellitus and is believed to play an important role in the development of diabetes-related complications [7]. Some studies have found that AOPP are moderately elevated in adult patients with Type 1 diabetes and more markedly elevated in those with Type 2 diabetes [8, 9], although others have suggested that AOPP are only significantly increased in Type 2 diabetes [10]. They have been reported to be elevated in Type 2 diabetes [11], and the concentration correlated with insulin resistance [12] or the presence and/or severity of diabetic complications [13, 14], such as retinopathy [15, 16] or nephropathy [17]. In juveniles and adolescents with Type 1 diabetes, AOPP were found to accumulate over time and were significantly associated with disease duration [18]. Others suggest that whilst AOPP concentrations are elevated in children with Type 1 diabetes, there is no association with disease duration [19].

We wished to adopt the AOPP assay for our own studies of diabetic patients and control subjects, examining the relationship between oxidative stress and risk factors such as BMI, waist : hip ratio, and blood pressure. However, we report here that the AOPP assay was prone to sample precipitation and consequent poor reproducibility, and we describe a novel modification of the method which measures the total iodide ion (I^−^) oxidising capacity of plasma (hereafter referred to as AOPP_TIOC_). The AOPP_TIOC_ assay eliminates interference by sample precipitation and provides greater reproducibility of measurements within samples and a more accurate determination of the ability of a plasma sample to oxidise I^−^ ions.

Application of this modified assay to plasma samples from human diabetic patients and nondiabetic control subjects demonstrated an increase in AOPP_TIOC_ with age in both diabetics and nondiabetics and a significant association with the presence of hypertension, although there was no difference in I^−^ oxidising capacity between the two groups.

## 2. Materials and Methods

### 2.1. Materials

KI, chloramine T hydrate, and PBS were all purchased from Sigma-Aldrich (Gillingham, UK). Glacial acetic acid was from Fisher Scientific (Loughborough, UK) and clear 96-well NUNC Maxisorp microplates were from Greiner Bio One (Stonehouse, UK).

### 2.2. Plasma Samples

Plasma samples from diabetic patients or nondiabetic controls were collected with informed consent by the NIHR Clinical Research Facility at Peninsula Medical School and analysed for their ability to oxidise I^−^ to I_3_^−^. This study had ethical approval from the North and East Devon Research Ethics Committee and was conducted according to the Declaration of Helsinki. A full medical history (including past medical history of myocardial infarction, peripheral vascular disease, hypertension, retinopathy, neuropathy, nephropathy, and cancer) plus physiological data (BMI, waist and hip measurements, and blood pressure) was taken from all participants at the time of venepuncture (Table 1). Routine clinical chemistry measures included triglycerides, HbA1c, and albumin. The mean age of the Type 2 diabetics was 64.0 ± 11.0 (mean ± 1 S.D.) years (*n* = 49), of the Type 1 or unclassified diabetics 32.3 ± 12.7 years (*n* = 3), and of the nondiabetic control subjects 57.8 ± 15.8 years (*n* = 38). Peripheral blood was collected into EDTA-coated tubes and centrifuged (3000 g, 10 min). Plasma was removed and dispensed into 100 *μ*L aliquots before storage at −80°C. Samples were thawed at room temperature before analysis, and repeated freeze-thaw cycles were avoided.

### 2.3. Previously Published AOPP Assay

For measurement of AOPP, plasma samples were diluted to 10% in PBS and 200 *μ*L applied in triplicate to a 96-well microplate. Standards of chloramine T (200 *μ*L; 0–100 *μ*M) were added to the plate. KI (1.16 M, 10 *μ*L) was added to all wells, followed 2 min later by bolus addition of 20 *μ*L glacial acetic acid. The optical density was then read immediately at 340 nm (OD_340_) using a Fluostar Optima plate reader (BMG Labtech Ltd., Aylesbury, UK).

### 2.4. AOPP_TIOC_ Assay

Measurement of AOPP_TIOC_ by our protocol was carried out as follows. Plasma samples were diluted to 10% in PBS and 300 *μ*L applied in triplicate to a 96-well microplate. Standards of chloramine T (300 *μ*L; 0–100 *μ*M) were added to the plate and a sample blank was prepared by adding 300 *μ*L PBS to the microplate. KI (1.16 M, 15 *μ*L) was added to all wells, followed 2 min later by bolus addition of 30 *μ*L glacial acetic acid. The microplate was centrifuged (5800 g, 5 min) to pellet precipitated protein, and 230 *μ*L of supernatant was transferred to a clean microplate. The OD_340_ of the supernatant was read 10 min after the addition of glacial acetic acid. The optical density at 595 nm (OD_595_), at which wavelength I_3_^−^ does not absorb [20], was also read as a “nonreaction” measure of sample turbidity and light scattering. The optical density of the sample blank was subtracted from all wells before data analysis.

### 2.5. Validation Studies

Three different dilutions (5%, 10%, and 20%) of three plasma samples selected at random were analysed for AOPP_TIOC_, followed by multiplication by the dilution factor, to determine whether the same value of AOPP_TIOC_ is obtained whatever the sample dilution.

Coefficients of variation were calculated between triplicate optical density measurements of a given sample (interreplicate CV), between AOPP_TIOC_ values calculated from numerous sets of triplicate measurements of the same sample on the same plate using the same standard curve (intra-assay CV), and between AOPP_TIOC_ values calculated from numerous sets of triplicate measurements of the same sample on different microplates using different standard curves (interassay CV).

### 2.6. Other Statistical Analyses

Statistical analyses were carried out using GraphPad Prism 5 statistical software package. Unpaired Student's *t*-test was used to evaluate differences in AOPP_TIOC_ values involving two sets of categorical data (diabetics versus nondiabetic controls; hypertensive versus nonhypertensive individuals; gender) and ANOVA was used where there were three or more sets of categorical data (smoking status). Linear regression analyses, using Pearson's rank correlation coefficients (*r*), were performed to assess associations of AOPP_TIOC_ values with continuous variables (age; diabetes disease duration; HbA1c, BMI; waist : hip ratio, serum albumin, and triglyceride concentration). In all cases, a *p* value <0.05 was taken to represent a statistically significant difference.

## 3. Results

Plasma was initially analysed using a previously published AOPP method [8–10, 12, 17]. Of the 77 plasma samples assessed from both diabetic patients and nondiabetic control subjects, only 27 produced an interreplicate CV of less than 20%, and only six of these were under 10% (see Supplementary Material 1 available online at http://dx.doi.org/10.1155/2015/496271). The median interreplicate CV of the 77 samples was 26.2% (interquartile range 16.7–44.0%). Consequently, high variability was seen when one sample was analysed on five separate occasions (Supplementary Material 2).

Precipitation was seen in plasma samples from both diabetics (31 of 39 samples; 79.5%) and control subjects (19 of 38 samples; 50.0%). The highest % CVs were seen in the samples that had lower blank optical density values (*r* = −0.310, *p* < 0.05), so the variability was not related to high inherent sample optical density; indeed the reverse was true.

**Figure 1:**
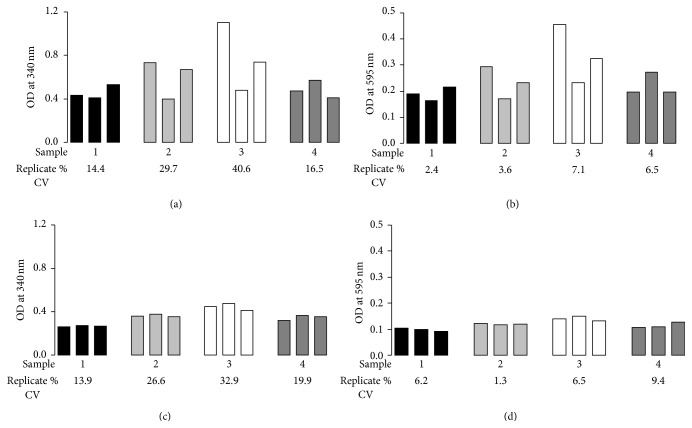
Improved reproducibility of the modified AOPP_TIOC_ assay after centrifugation (5800 g, 5 min) of the microplate and transfer of supernatant. Four random plasma samples were taken and optical density was measured at a wavelength of 340 nm following (a) the original protocol and (c) our modified protocol. Optical density was also measured at 595 nm in (b) the original protocol and (d) our modified protocol in order to examine sample turbidity. Bars represent triplicate optical density values obtained, with each type of shading representing a different plasma sample. Numbers under the appropriate bars represent the % coefficient of variation (% CV) of the triplicate measurements for that particular sample.

The individual microplate wells that gave a high apparent optical density reading corresponded to wells in which a precipitate could be seen. Furthermore, the change in OD_595_ on addition of I^−^ and acetic acid mirrored the change in OD_340_ (representative data from four samples is shown in Figure 1, panels (a) and (c)), demonstrating that sample turbidity had altered and variability between replicate values was not alone due to differences in the extent of I^−^ oxidation. Therefore the assay was modified in order to allow the removal of the precipitate from the samples before the optical density was read, to generate a method which we named “AOPP_TIOC_.” This involved increasing all volumes in the assay by 50%, followed by centrifugation of the microplate (5800 g, 5 min) and the subsequent transfer of supernatants (230 *μ*L, equal to the final volume per well in the original method) to a fresh microplate before optical density measurements were taken. The increase in sample and reagent volumes by 50% facilitated the removal of the supernatant after microplate centrifugation, leaving behind the pelleted precipitate, which was discarded.

Four plasma samples were selected to test the reproducibility of replicates using this modified method. The same samples were also analysed by the original method for comparison. Using the original method, interreplicate CVs were all over 10% (range 14.4–40.6%; Figure 1(a)) and nonreaction measurements (OD_595_) were similarly variable (range 13.9–32.9%; Figure 1(c)). In contrast, using our modified protocol, optical density measurements were lower than those seen with the original method and more consistent, with interreplicate CVs all under 10% (range 2.4–7.1%; Figure 1(b)). The improved reproducibility of these measurements was mirrored by the low variability in optical density values at 595 nm (range 1.3–9.4%; Figure 1(d)), indicating that the precipitate had successfully been removed.

Three plasma samples were selected and diluted to 5%, 10%, and 20% in PBS. AOPP_TIOC_ was then measured using our method and the values obtained multiplied by the appropriate dilution factor for comparison purposes (Table 2, column 6). Although the optical density of the standards was linear across the range of concentrations tested as previously described [2], and blank OD values of plasma samples (before addition of I^−^ and acetic acid; Table 2, column 3) also approximated the expected linear relationship, this was not the case for plasma samples following addition of iodide and acid. As the amount of plasma present in the well doubled (20% versus 10% plasma and 10% versus 5% plasma), the associated AOPP_TIOC_ increased fourfold. Similarly, a fourfold increase in plasma concentration (20% versus 5% plasma) produced an AOPP_TIOC_ value that was sixteen times higher. This could be converted to a linear relationship by a square root transformation of AOPP_TIOC_ calculated from the standard curve as chloramine T equivalents. Doing this, and subsequently multiplying by the dilution factor, produced consistent AOPP_TIOC_ values. The mean ± S.D. of the AOPP_TIOC_ concentration determined from the three different dilutions was 42.5 ± 1.6 *μ*M in one plasma sample, 46.0 ± 0.3 *μ*M in another, and 53.6 ± 3.0 *μ*M in the third (Table 2, column 8).

This relationship between plasma dilution and OD_340_ does not merely represent differences in the degree of light scattering due to protein precipitation. Actual fold-change in OD at a nonspecific wavelength (OD_595_), at which I_3_^−^ does not absorb light, did not show the same relationship between OD and plasma dilution (data not shown). Thus, association of OD with the square of the plasma sample concentration was specific for the 340 nm wavelength at which I_3_^−^ absorbs light.

It was noted that, despite the centrifugation step, there remained a difference in OD_595_ between samples (typically ranging from 0.05 to 0.15 OD; data not shown) and within the same sample analysed on different days. These differences probably reflect inherent differences in sample composition that are present even before the initiation of protein precipitation and varying degrees of precipitate formation and removal from one sample on different days, respectively. Such differences may interfere with the assay and make comparisons between samples problematic. Therefore, OD_340_ was additionally normalised to an OD_595_ of 0.1, in order to account for these differences, and the resulting normalised OD_340_ value (nOD_340_) was analysed by linear regression using the standard curve.

Taking the above two points into consideration, we derived the following algorithm for the determination of AOPP_TIOC_.

**Step 1 .**

Calculation of normalised OD_340_ (nOD_340_) is as follows:

(1) $$\begin{array}{c} {\text{nOD}}_{340}=\left[\;\frac{\left({\text{OD}}_{340}\right)}{\left({\text{OD}}_{595}\times 10\right)}\;\right]. \end{array}$$

**Step 2 .**

Calculation of *x* from nOD_340_ using linear regression is as follows:

(2) $$\begin{array}{c} x=\frac{\left({\text{nOD}}_{340}-c\right)}{m}, \end{array}$$

where *y* = *mx* + *c* describes the equation of the standard curve and *y* = nOD_340_.

**Step 3 .**

Square root transformation of *x*  (*x*_srt_) is as follows:

(3) $$\begin{array}{c} x_{\text{srt}}=\left(\;x^{1/2}\;\right). \end{array}$$

**Step 4 .**

Multiplication by dilution factor is as follows:

(4) AOPPTIOC=xsrt×10μM  chloramine  T  equivalents.

The intra-assay and interassay reproducibility of the assay were assessed by measuring AOPP_TIOC_ as described above in several plasma samples. AOPP_TIOC_ was measured six times in triplicate on the same plate in five plasma samples selected at random (Samples A–E).  Table 3 shows that the intra-assay CV in all five samples was under 6% (1.7–5.3%). Interassay CV determinations were carried out using five plasma samples, each of which was measured at least six times over a period of 14 months (Samples F and G) or four months (Samples H–J). Interassay CVs for the five samples ranged from 5.3 to 10.5%, as shown in Table 3.

**Figure 2:**
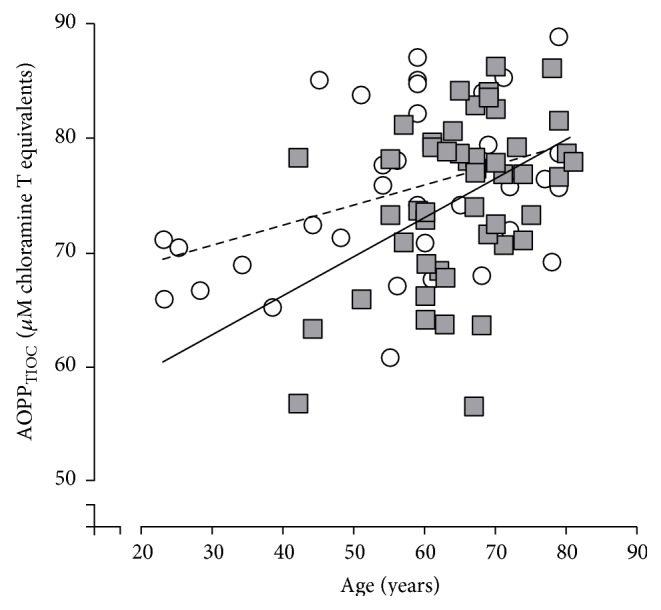
Association of AOPP_TIOC_ with age in nondiabetic and diabetic subjects. AOPP_TIOC_ was measured in 37 subjects without diabetes and 50 patients with Type 2 diabetes and linear regression analyses (Pearson's rank correlation) of AOPP_TIOC_ versus age were performed. Both diabetic (*r* = 0.485) and nondiabetic (*r* = 0.388) individuals showed a significant (*p* < 0.05) increase in AOPP_TIOC_ with age. Open circles represent data from nondiabetic individuals and dashed line shows the linear trendline through the data. Grey squares represent subjects with Type 2 diabetes and the linear trendline is shown by the solid line.

The modified assay was then applied to 90 plasma samples from individuals with diabetes or control subjects (50 Type 2 diabetics, 3 subjects with unclassified diabetes, and 37 nondiabetic controls). It was found that AOPP_TIOC_ values were not significantly different between Type 2 diabetic and nondiabetic individuals (*p* = 0.665 by unpaired Student's *t*-test). Mean (± 1 S.D.) AOPP_TIOC_ in diabetics was 74.8 ± 7.2 *μ*M chloramine T equivalents and in controls was 75.5 ± 7.0 *μ*M chloramine T equivalents. It was found that AOPP_TIOC_ values associated significantly with age in both nondiabetic (*r* = 0.392, *p* < 0.05) and Type 2 diabetic (*r* = 0.489, *p* < 0.05) subjects, as assessed by Pearson's rank correlation (Figure 2), although AOPP_TIOC_ did not correlate with disease duration in 46 Type 2 diabetes patients for whom this information was available (*r* = −0.024; *p* > 0.05). AOPP_TIOC_ also significantly associated with age in the combined dataset of 90 subjects, including the three subjects with Type 1 or unclassified diabetes (*r* = 0.393, *p* < 0.05).

**Figure 3:**
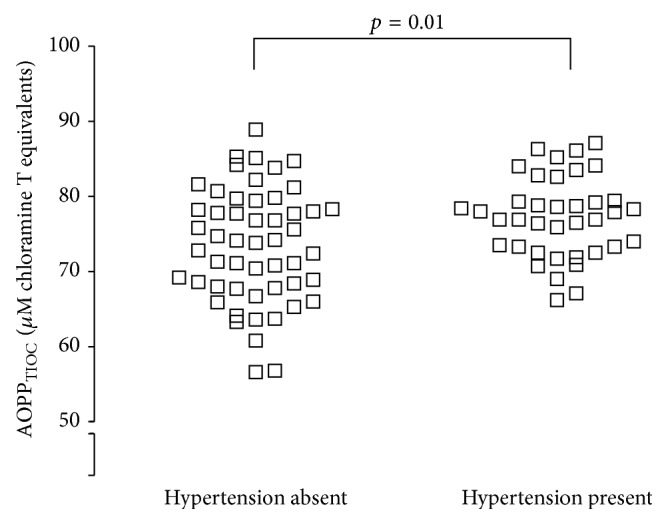
Elevated AOPP_TIOC_ in subjects with hypertension. Subjects were divided into 52 normotensive individuals and 38 subjects diagnosed with hypertension and logistic analysis of their associated plasma AOPP_TIOC_ concentrations performed by two-tailed Student's *t*-test. A significant (*p* < 0.05) difference in AOPP_TIOC_ was found between the two groups.

Data from all 90 subjects was analysed in relation to other morbidities that may influence AOPP_TIOC_ values, in particular those that increase in prevalence with age. Using this approach, only hypertension (38 hypertensive subjects and 52 nonhypertensive subjects) was present in sufficient numbers of subjects to allow valid analyses to be undertaken. It was found that AOPP_TIOC_ associates with hypertension (*p* = 0.01 by two-tailed unpaired Student's *t*-test), regardless of whether or not the subjects had diabetes (Figure 3). Although the maximum values were similar in nonhypertensive (88.9 *μ*M chloramine T equivalents) and hypertensive (87.1 *μ*M chloramine T equivalents) subjects, the data in hypertensive individuals was clustered around the higher end of the range of values such that the minimum value in hypertensives was 67.1 *μ*M chloramine T equivalents compared with 56.6 *μ*M chloramine T equivalents in nonhypertensives. The mean age of the hypertensive individuals (66.8 ± 1.3 years) was significantly higher (*p* < 0.001, two-tailed unpaired Student's *t*-test) than the nonhypertensive individuals (55.6 ± 2.3 years).

No association was seen between AOPP_TIOC_ concentration and BMI (*n* = 90, *r* = 0.085; *p* > 0.05) as assessed by Pearson's rank correlation analysis. A trend towards an association with waist:hip ratio was seen, but this did not reach statistical significance in this study (*n* = 87, *r* = 0.195; *p* > 0.05). Furthermore, there was no correlation between AOPP_TIOC_ and HbA1c (*n* = 36, *r* = −0.138; *p* > 0.05), serum albumin (*n* = 29; *r* = −0.084; *p* > 0.05), or triglycerides (*n* = 27, *r* = −0.187; *p* > 0.05).

Smoking status did not influence the values obtained, as mean ± 1 S.D. AOPP_TIOC_ was 75.5 ± 6.5 *μ*M chloramine T equivalents in nonsmokers (*n* = 44), 74.6 ± 7.6 *μ*M chloramine T equivalents in past smokers (*n* = 39), and 74.4 ± 7.5 *μ*M chloramine T equivalents in present smokers (*n* = 7) (*p* > 0.05 by unpaired one-way ANOVA). Additionally, there was no difference between males (74.7 ± 7.3 *μ*M chloramine T equivalents, *n* = 46) and females (75.5 ± 6.7 *μ*M chloramine T equivalents, *n* = 44) as assessed by unpaired Student's *t*-test.

A subset of 23 samples was reanalysed between nine and 22 months after initial analysis in order to determine the reproducibility of incurred samples on repeat analysis. Twenty-two of these samples (95.7%) were found to give AOPP concentrations within 20% of the original values, and 52.2% of samples were within 10%.

## 4. Discussion

In the present study, we set out to study oxidative stress in patients with diabetes. We used the AOPP assay, in which the capacity of plasma samples to oxidise I^−^ to I_3_^−^ is analysed [16, 17, 21–25]. Chloramine T is used as a standard, although a number of plasma constituents contribute to the oxidation of I^−^ to I_3_^−^, including hydroperoxides [26], peroxidases [27, 28], and dehydromethionine [29]. The chemical equation of the reaction between chloramines and I^−^ is

(5) RNHCl+3I−colourless+H+⟷I3−yellow-brown+RNH2+Cl−

The I_3_^−^ product has a *λ*_max⁡_ at 353 nm [20] and can therefore be measured spectrophotometrically. Recent studies [21, 30] have suggested that oxidised fibrinogen is the oxidiser of I^−^ in this assay, where oxidised fibrinogen may, in turn, result from the activity of myeloperoxidase during inflammation [31]. However, this method showed very large discrepancies between replicate measurements for plasma samples, due to previously unreported interference by a protein precipitate, which varied between wells and between samples. Therefore, in its existing form, the AOPP assay was unsuitable for the reliable determination of AOPP.

We set out to modify the protocol to generate a method that we termed AOPP_TIOC_ such that the interreplicate CV was reduced and the intra- and interassay CVs were minimized [32]. It was necessary to remove the precipitate formed on addition of I^−^ and acetic acid by microplate centrifugation to pellet the precipitate, followed by transfer of supernatant to a new microplate before measurement. The OD_340_ of triplicate measurements then became acceptably reproducible. There was no correlation between AOPP_TIOC_ concentration and serum albumin concentration, demonstrating that the modified method is independent of protein concentration and does not merely reflect increased precipitation in samples with higher protein content.

Reproducibility studies showed the modified assay to be within acceptable limits. Interassay variation was not higher in those samples in which repeat analyses were performed over a longer period of time (14 months) compared to a short period of time (4 months), suggesting that AOPP are stable on storage at −80°C for at least one year. Incurred sample reanalysis (ISR) surpassed the recommended standards for analytical methods. The Crystal City III guidelines issued by the FDA in 2007 recommended that two-thirds of samples must fall within 20% of the original value on ISR in order for an analytical assay to be deemed valid [33].

We wished to establish if the modified AOPP_TIOC_ protocol also demonstrated increased values in diabetic patients. However, no such differences existed between the populations of diabetics and nondiabetics, and there was no association with poor glycaemic control, as measured by glycated haemoglobin (HbA1c).

However, AOPP_TIOC_ was significantly associated with the presence of hypertension, a common comorbidity of diabetes. A previous study reported that AOPP concentrations correlate with the degree of arterial stiffness in humans [24], which leads to an elevation of systolic blood pressure and risk of clinical hypertension [34]. Furthermore, it has recently been reported that AOPP is associated with endothelial dysfunction [35] which predisposes to hypertension. Both the above studies involved addition of KI to samples as well as to standards. Thus, our findings support the conclusions of these previous studies which used a method that is closely related to our modified version.

Our inability to reproduce previous studies examining AOPP concentrations in diabetics and nondiabetic controls might be explained by a higher incidence of hypertension in the diabetic cohort of other studies, as hypertension is a common comorbidity of diabetes. Hypertension (or subclinical elevated blood pressure) rather than diabetes* per se* may have been the factor linked to increased iodide oxidation in the diabetic group. For example, in one study [17], mean blood pressure (systolic ± 1 S.D./diastolic ± 1 S.D.) in healthy controls was 118 ± 29/72 ± 19 mm Hg and in diabetics was 141 ± 14/76 ± 10 mm Hg. Additionally, Kalousová et al. [8] reported that AOPP in patients with Type 2 diabetes (mean blood pressure 139 ± 9/86 ± 6 mm Hg) was more highly elevated from healthy control values (*p* < 0.001) than in patients with Type 1 diabetes (mean blood pressure 123 ± 13/80 ± 7 mm Hg; *p* < 0.05 versus healthy controls). Thus, the patient group with a more strongly elevated mean blood pressure was also the group which showed a higher elevation of mean AOPP concentration.

Alternatively, interference in the AOPP assay by elevated triglycerides [36] due to hyperlipidaemia or differences in fasting status between sample groups may account in part for the previous observations. Triglycerides are carried in the blood in the form of chylomicrons, which scatter light [37], but our modification of the protocol accounts for differences in light scattering by normalising for the optical density of the sample at 595 nm. In one previously published study, the mean triglyceride concentration in healthy controls was 102.7 ± 48.5 mg/dL compared to 170.0 ± 71.3 mg/dL in diabetics [17]. Another study reported that AOPP was higher in Type 2 diabetes (2.1 ± 1.0 mM triglycerides) than Type 1 diabetes (1.2 ± 0.5 mM) [8]. In a further study, AOPP were reported to be elevated in diabetes (0.84 ± 0.07 mM triglycerides), particularly in the presence of complications (1.17 ± 0.14 mM) compared to controls (0.63 ± 0.04 mM) [38]. In our study, no association was found between triglyceride concentration and AOPP_TIOC_ concentration, suggesting that our modification of the assay is not subject to interference by triglycerides as is the case for other versions of the assay. Similarly, other plasma components, such as NADH and NADPH, absorb light at 340 nm [39] and may interfere with an assay which only measures the optical density of a plasma sample at this wavelength. However, no association was seen between AOPP_TIOC_ concentration and blank OD_340_ in all subjects assessed during this study. This suggests that our assay is independent of the NADH and NADPH concentration of the sample.

The Free Radical Theory of Ageing postulates that the progressive loss of function observed in the ageing process is due to an accumulation of damage to cellular components by oxidant species [40]. Few studies have thus far examined the validity of this theory in humans, and this is largely due to the lack of robust assays suitable for high-throughput analysis. The oxidising capacity of plasma, as measured by our modified AOPP assay, increased with age in the sample population of 90 subjects, supporting previous findings of an accumulation of oxidative stress in human ageing by other groups [41–47]. It should be noted, however, that the prevalence of hypertension increases with advancing age, and the cause-effect relationship of AOPP in ageing and hypertension has not yet been established. Further studies would be required to determine whether AOPP accumulate with age and predispose to hypertension, or whether AOPP are markers of hypertension that becomes more likely in older individuals.

AOPP have been widely accepted into the field of oxidative stress research, as a marker of plasma protein oxidative damage [8–10, 12, 15, 17, 18, 22, 23, 31, 35, 38, 41, 44, 48–51]. However, on thorough study of the literature, it is apparent that two discrete versions of the AOPP assay exist which both, at first sight, resemble an assay to measure chloramines, in which the oxidation of iodide (I^−^) to triiodide (I_3_^−^) by phagocyte-derived chloramines is measured [52]. It is clear that the analytes measured in the two versions of the AOPP assay are different, but this discrepancy has not previously been pointed out.

In one version of the AOPP assay, optical density of the plasma sample at 340 nm is simply measured in the presence of acid [4, 5, 36, 48, 49] and is apparently based on the rationale that oxidatively modified proteins absorb light at 340 nm to a greater extent than native proteins under acidic conditions [53]. The relevance of using chloramine T as a standard [2, 36, 49, 50] is not made clear. In this version of the assay, the measured AOPP are reported to be carried largely by oxidised albumin and are made up of numerous chromophores including dityrosine, pentosidine, and protein carbonyls [53], but AOPP themselves have no measured oxidising capacity. The second version of the method examines the iodide oxidising capacity of plasma, and this is the principle of the assay developed in the present study [8–10, 12, 17]. Using this version of the assay, AOPP are reported to comprise oxidised fibrinogen [21, 30].

AOPP have previously been reported to be elevated in both Type 1 and Type 2 diabetic patients compared with healthy controls, but some studies were carried out using the AOPP protocol in which KI is only added to the standards [38] and others examining plasma iodide oxidising capacity [8–10, 12, 17]. In yet further studies on AOPP in diabetes, it was unclear which protocol was followed [15, 18, 51]. Differences in methodology may partially explain the discrepancy in AOPP values published in previous studies of oxidative stress. Reported AOPP concentrations in control groups vary greatly between studies, with concentrations ranging from 0.11 ± 0.05 [17] to 259 ± 75 *μ*M [12] chloramine T equivalents, and on examination of the two versions of the assay it appears that the two published AOPP methods measure different analytes. Additionally, failure to correct for precipitation or inherent triglyceride concentrations may influence AOPP concentrations determined during these studies. The availability of a robust assay of AOPP_TIOC_ will facilitate further studies to characterise the components responsible for plasma TIOC activity.

In summary, we have identified inconsistencies in the published protocols by which AOPP is measured, which have not previously been highlighted. One such method is confounded by precipitation which causes variability between replicates and overestimation of AOPP concentrations. We have developed a modified version of this method (named AOPP_TIOC_) which eliminates the effect of precipitation and has been shown to increase with age in human plasma and is significantly associated with hypertension, suggesting that it may be a marker of endothelial dysfunction. We strongly urge other researchers wishing to publish AOPP data to be particularly careful in the description of their methodology, especially with regard to whether or not I^−^ is added to plasma samples as well as to standards.

## Supplementary Material

Supplementary Material 1: Optical density readings at 340 nm (OD340) of 77 human plasma samples, analysed in triplicate using the previously-published AOPP assay protocol, with associated means, standard deviations (S.D.) and coefficients of variation (% CV = S.D./mean x 100%) of the triplicate measurements for each sample." etc.
Supplementary Material 2: Results from triplicate determinations of one plasma sample performed in 5 runs of the AOPP assay. " etc.

## Figures and Tables

**Table 1:** Demographic characteristics of the study cohort.

	Controls	T2DM	T1/unclassified DM	Total
*n *	38	49	3	90
Age (years ± S.D.)	57.8 ± 15.8	64.0 ± 11.0	32.3 ± 12.7	60.3 ± 14.6
Gender (male/female)	13/25	32/17	1/2	46/44
Smoking status (present/past/never)	4/11/23	3/27/19	0/1/2	7/39/44
Hypertension (absent/present)	26/12	23/26	3/0	52/38
BMI (mean ± S.D.)	27.2 ± 5.4	29.7 ± 5.3	25.2 ± 0.28	28.7 ± 5.4
Waist : hip (mean ± S.D.)	0.87 ± 0.10	0.96 ± 0.09	0.81 ± 0.11	0.92 ± 0.10

**Table 2:** Evaluation of the linearity of AOPP_TIOC_ measurements.

Plasma sample number	Dilution (%)	Mean blank	Mean test	AOPP_TIOC_ (*μ*M chloramine T equivalents)			
		OD_340_	OD_340_	AOPP_TIOC_1^a^	x dilution	AOPP_TIOC_2^b^	x dilution
1	5	0.068	0.032	4.37	**87.4**	2.09	**41.8**
	10	0.123	0.131	17.17	**171.7**	4.14	**41.4**
	20	0.209	0.602	78.38	**391.9**	8.85	**44.3**

2	5	0.075	0.040	5.35	**107.1**	2.31	**46.3**
	10	0.130	0.161	21.07	**210.7**	4.59	**45.9**
	20	0.224	0.641	83.46	**417.3**	9.14	**45.7**

3	5	0.123	0.058	7.76	**155.2**	2.79	**55.7**
	10	0.220	0.202	26.48	**264.8**	5.15	**51.5**
	20	0.404	0.800	104.13	**520.6**	10.20	**51.0**

OD_340_: optical density at 340 nm. ^a^AOPP_TIOC_1: AOPP_TIOC_ concentration calculated from original optical density values using the standard curve. ^b^AOPP_TIOC_2: square root of AOPP_TIOC_1.

**Table 3:** Intra-assay and interassay coefficients of variation.

AOPP_TIOC_ values (*μ*M chloramine T equivalents)					
Intra-assay	Sample A (*n* = 6)	Sample B (*n* = 6)	Sample C (*n* = 6)	Sample D (*n* = 6)	Sample E (*n* = 6)

Mean	77.6	79.7	70.2	56.7	57.6
S.D.	1.3	2.7	3.4	2.0	3.1
CV (%)	**1.7**	**3.4**	**4.8**	**3.5**	**5.3**

Interassay	Sample F (*n* = 6)	Sample G (*n* = 6)	Sample H (*n* = 8)	Sample I (*n* = 7)	Sample J (*n* = 8)

Mean	74.6	68.9	65.3	72.5	73.2
S.D.	5.2	3.6	6.8	6.7	6.7
CV (%)	**7.0**	**5.3**	**10.5**	**9.2**	**9.2**

## Abbreviations

AOPP: Advanced oxidation protein products
BMI: Body mass index
CV: Coefficient of variation
KI: Potassium iodide
OD: Optical density
PBS: Phosphate buffered saline
TIOC: Total iodide oxidising capacity.
