# Supplementary material for: Optimisation of an Advanced Oxidation Protein Products Assay: Its Application to Studies of Oxidative Stress in Diabetes Mellitus
Source: Oxid Med Cell Longev. 2015 May 14;2015:496271. doi: 10.1155/2015/496271 (PMC4465816; doi:10.1155/2015/496271)
Supplement: Supplementary file 1 — Supplementary Material 1: Optical density readings at 340 nm (OD340) of 77 human plasma samples, analysed in triplicate using the previously-published AOPP assay protocol, with associated means, standard deviations (S.D.) and coefficients of variation (% CV = S.D./mean x 100%) of the triplicate measurements for each sample." etc. Supplementary Material 2: Results from triplicate determinations of one plasma sample performed in 5 runs of the AOPP assay. " etc. [file 496271.f1.pdf]

Sample	Type	OD$_{340}$ measurements			Mean	S.D.	% CV
		**1**	**2**	**3**			
1	C	0.524	0.237	0.418	0.393	0.145	**36.9**
2	C	0.599	0.368	0.743	0.570	0.189	**33.2**
3	C	0.199	0.223	0.181	0.201	0.021	**10.5**
4	C	1.076	0.588	0.663	0.776	0.263	**33.9**
5	C	0.292	0.334	0.281	0.302	0.028	**9.3**
6	DM	0.835	0.274	0.273	0.461	0.324	**70.4**
7	C	0.326	0.451	0.365	0.381	0.064	**16.8**
8	DM	0.598	0.350	0.616	0.521	0.149	**28.5**
9	C	0.513	0.378	0.443	0.445	0.068	**15.2**
10	DM	0.340	0.486	0.328	0.385	0.088	**22.9**
11	DM	0.325	0.574	0.267	0.389	0.163	**42.0**
12	DM	0.400	0.306	0.256	0.321	0.073	**22.8**
13	DM	0.358	0.343	0.306	0.336	0.027	**8.0**
14	C	0.708	0.606	0.412	0.575	0.150	**26.1**
15	DM	0.725	0.655	0.480	0.620	0.126	**20.4**
16	C	0.372	0.829	0.330	0.510	0.277	**54.2**
17	C	0.694	0.898	0.858	0.817	0.108	**13.2**
18	C	0.638	0.590	0.679	0.636	0.045	**7.0**
19	DM	0.761	0.923	0.719	0.801	0.108	**13.4**
20	DM	0.883	0.401	0.512	0.599	0.252	**42.2**
21	C	0.667	0.663	0.902	0.744	0.137	**18.4**
22	C	0.488	0.469	0.772	0.576	0.170	**29.4**
23	C	0.903	0.871	1.067	0.947	0.105	**11.1**
24	C	0.749	0.465	0.993	0.736	0.264	**35.9**
25	C	0.960	0.850	1.190	1.000	0.173	**17.3**
26	C	1.072	0.625	0.953	0.883	0.232	**26.2**
27	C	0.661	0.857	0.692	0.737	0.105	**14.3**
28	DM	0.543	0.561	1.065	0.723	0.296	**41.0**
29	DM	0.915	0.703	0.375	0.664	0.272	**41.0**
30	DM	0.637	0.371	0.352	0.453	0.159	**35.1**
31	DM	1.031	0.402	0.738	0.724	0.315	**43.5**
32	DM	0.843	0.733	0.880	0.819	0.076	**9.3**
33	DM	0.484	0.513	0.649	0.549	0.088	**16.1**
34	DM	0.592	1.245	1.114	0.984	0.345	**35.1**
35	DM	0.495	1.349	1.094	0.979	0.438	**44.8**
36	DM	0.506	1.155	1.129	0.930	0.367	**39.5**
37	DM	0.627	0.313	0.307	0.416	0.183	**44.0**
38	DM	0.998	0.512	0.449	0.653	0.300	**46.0**
39	DM	1.261	1.002	1.387	1.217	0.196	**16.1**
40	C	1.040	0.704	0.777	0.840	0.177	**21.0**
41	DM	0.644	0.995	0.814	0.818	0.176	**21.5**
42	DM	1.244	0.761	1.067	1.024	0.244	**23.9**
43	C	0.620	0.841	0.681	0.714	0.114	**16.0**
44	C	1.311	0.379	0.269	0.653	0.572	**87.7**
45	DM	0.558	0.382	0.380	0.440	0.102	**23.2**
46	C	0.298	0.617	0.250	0.388	0.199	**51.4**
47	DM	0.288	0.268	0.337	0.298	0.036	**11.9**
48	DM	1.082	0.591	1.158	0.944	0.308	**32.6**
49	DM	0.786	0.689	0.529	0.668	0.130	**19.4**

50	C	0.355	0.962	0.325	0.547	0.359	**65.7**
51	C	0.308	0.270	1.453	0.677	0.672	**99.3**
52	DM	0.527	0.215	0.233	0.325	0.175	**53.9**
53	DM	1.461	0.947	1.137	1.182	0.260	**22.0**
54	C	1.028	0.236	0.327	0.530	0.433	**81.7**
55	DM	0.765	0.286	0.293	0.448	0.275	**61.3**
56	DM	1.331	0.281	0.405	0.672	0.574	**85.3**
57	C	1.155	1.335	0.241	0.910	0.587	**64.4**
58	DM	0.702	0.824	0.269	0.598	0.292	**48.7**
59	DM	0.374	0.210	0.565	0.383	0.178	**46.4**
60	C	1.186	1.351	0.936	1.158	0.209	**18.0**
61	C	0.319	0.325	0.313	0.319	0.006	**1.9**
62	DM	0.373	0.274	0.276	0.308	0.057	**18.4**
63	DM	0.619	1.006	0.975	0.867	0.215	**24.8**
64	DM	0.692	0.367	0.627	0.562	0.172	**30.6**
65	DM	0.487	0.945	1.350	0.927	0.432	**46.6**
66	C	0.465	0.326	0.443	0.411	0.075	**18.2**
67	C	0.361	0.397	0.367	0.375	0.019	**5.1**
68	C	0.272	0.750	1.817	0.946	0.791	**83.6**
69	DM	0.605	0.510	1.202	0.772	0.375	**48.6**
70	DM	0.616	0.736	1.003	0.785	0.198	**25.2**
71	C	1.363	1.817	1.878	1.686	0.281	**16.7**
72	C	1.008	1.057	1.220	1.095	0.111	**10.1**
73	C	1.051	1.271	2.025	1.449	0.511	**35.3**
74	C	1.317	1.389	1.709	1.472	0.209	**14.2**
75	C	0.570	1.321	1.956	1.282	0.694	**54.1**
76	C	0.452	0.834	1.171	0.819	0.360	**43.9**
77	C	1.158	1.324	1.462	1.315	0.152	**11.6**

Supplementary Material 1: Optical density readings at 340 nm (OD_{340}) of 77 human plasma samples, analysed in triplicate using the previously-published AOPP assay protocol , with associated means, standard deviations (S.D.) and coefficients of variation (% CV = S.D./mean x 100%) of the triplicate measurements for each sample. C = plasma sample from a control (non-diabetic) subject, DM = plasma sample from a diabetic individual.

	OD repl1	OD repl2	OD repl3	OD mean	Chl-T equivs (µM)	S.D.	% CV
**Assay 1**	0.537	0.360	0.512	0.470	256	0.096	20.4
**Assay 2**	0.483	0.456	0.459	0.466	253	0.015	3.1
**Assay 3**	0.405	1.329	1.323	1.019	651	0.532	52.2
**Assay 4**	0.406	0.725	0.705	0.612	358	0.178	29.2
**Assay 5**	0.486	0.501	1.829	0.939	593	0.771	82.1

Supplementary Material 2: Results from triplicate determinations of one plasma sample performed in 5 runs of the AOPP assay. The mean chloramine-T equivalent value of these five measurements was 422 µM with a % CV of 44.6.

OD = optical density at 340 nm; Chl-T equivs = chloramine T equivalents; "repl 1" = replicate 1; mean = mean OD of triplicate measurements; S.D. = standard deviation of triplicate measurements; % CV = % CV of triplicate measurements.
